# The artificial oxygen carrier erythrocruorin—characteristics and potential significance in medicine

**DOI:** 10.1007/s00109-023-02350-3

**Published:** 2023-07-18

**Authors:** Weronika Kruczkowska, Mateusz Kciuk, Zbigniew Pasieka, Karol Kłosiński, Elżbieta Płuciennik, Jacob Elmer, Klaudia Waszczykowska, Damian Kołat, Żaneta Kałuzińska-Kołat

**Affiliations:** 1 Faculty of Biomedical Sciences, Medical University of Lodz, Zeligowskiego 7/9, 90-752, Lodz, Poland; 2 Department of Molecular Biotechnology and Genetics, University of Lodz, Banacha 12/16, 90-237, Lodz, Poland; 3 Doctoral School of Exact and Natural Sciences, University of Lodz, Banacha 12/16, 90-237, Lodz, Poland; 4 Department of Experimental Surgery, Medical University of Lodz, Narutowicza 60, 90-136, Lodz, Poland; 5 Department of Functional Genomics, Medical University of Lodz, Zeligowskiego 7/9, 90-752, Lodz, Poland; 6 Department of Chemical and Biological Engineering, Villanova University, Villanova, PA USA

**Keywords:** Erythrocruorin, Hemoglobin, Artificial oxygen carriers, Red blood cell substitutes

## Abstract

The diminishing supply and increasing costs of donated blood have motivated research into novel hemoglobin-based oxygen carriers (HBOCs) that can serve as red blood cell (RBC) substitutes. HBOCs are versatile agents that can be used in the treatment of hemorrhagic shock. However, many of the RBC substitutes that are based on mammalian hemoglobins have presented key limitations such as instability and toxicity. In contrast, erythrocruorins (Ecs) are other types of HBOCs that may not suffer these disadvantages. Ecs are giant metalloproteins found in annelids, crustaceans, and some other invertebrates. Thus far, the Ecs of *Lumbricus terrestris* (LtEc) and *Arenicola marina* (AmEc) are the most thoroughly studied. Based on data from preclinical transfusion studies, it was found that these compounds not only efficiently transport oxygen and have anti-inflammatory properties, but also can be modified to further increase their effectiveness. This literature review focuses on the structure, properties, and application of Ecs, as well as their advantages over other HBOCs. Development of methods for both the stabilization and purification of erythrocruorin could confer to enhanced access to artificial blood resources.

**Supplementary Information:**

The online version contains supplementary material available at 10.1007/s00109-023-02350-3.

## Introduction—motivation for the development of novel RBC substitutes

Transfusion is a standard procedure that allows the substitution of the missing blood components. The initial issue with this procedure is related to the availability of donors [1, 2], especially for individuals with a rare blood group. According to statistics, only 7% of the general population are donors with universal blood (type “O” and rhesus negative [Rh] group) [3]. Secondly, an aging society could lead to a decrease in potential donors in 10–20 years [4]. The third issue is the rising expense of donor blood collection due to the expanded screening for blood-borne viruses such as the Zika virus or human immunodeficiency virus (HIV) [1]. Finally, it is important to note that RBCs have a limited half-life (42 days under refrigeration) [2, 3, 5] and that they can develop significant storage lesions after 48 h (e.g., stiffness of the RBC membrane or oxidation of the intracellular hemoglobin) [6]. It is urgent to increase the usage of RBC substitutes, also known as artificial oxygen carriers, that may be used for trauma patients who have sudden or unexpected blood loss [7]. They provide the possibility of oxygen treatments in diseases characterized by an absence of oxygen [1, 3, 5]. Additionally, the acellular red blood cell substitutes could tackle the problem of blood group matching [8] that would be beneficial during surgeries or cancer chemotherapy [7, 9, 10]. Studies on various organs, including the liver or kidney, have revealed that hemoglobin-based oxygen carriers (HBOCs) can also be employed for the reperfusion and maintenance of organs that are suitable as transplants [11–13]. Using PubMed, Science Direct, and Web of Science databases, in this paper we reviewed the RBC substitutes-related data published mainly from 2000 to the present using keywords “red blood cell substitutes,” “hemoglobin,” “erythrocytes,” “artificial oxygen carriers,” with emphasis on one of the promising artificial oxygen carriers, i.e., “erythrocruorin.”

### Mechanism of action of red blood cell substitutes (with examples)

Understanding red blood cell substitutes’ mechanism of action is crucial for their clinical advancement. Hemoglobin is a tetrameric protein found in RBCs that contributes to their characteristic red color [14]. Owing to Hb, red blood cells can bind oxygen in the lungs and transport it to other tissues/organs. This protein also allows the transport of CO_2_ (approx. 30%) in the opposite direction together with the products of cell metabolism from tissues and organs. Moreover, hemoglobin is referred to as a protein buffer because it maintains the proper pH of the plasma. Hb is composed of two α and two β polypeptide chains [14, 15]. Each of them has a heme group containing an iron ion. One heme group is capable of binding one O_2_ molecule, which allows one Hb molecule to carry four O_2_ molecules [5].

**Fig. 1:**
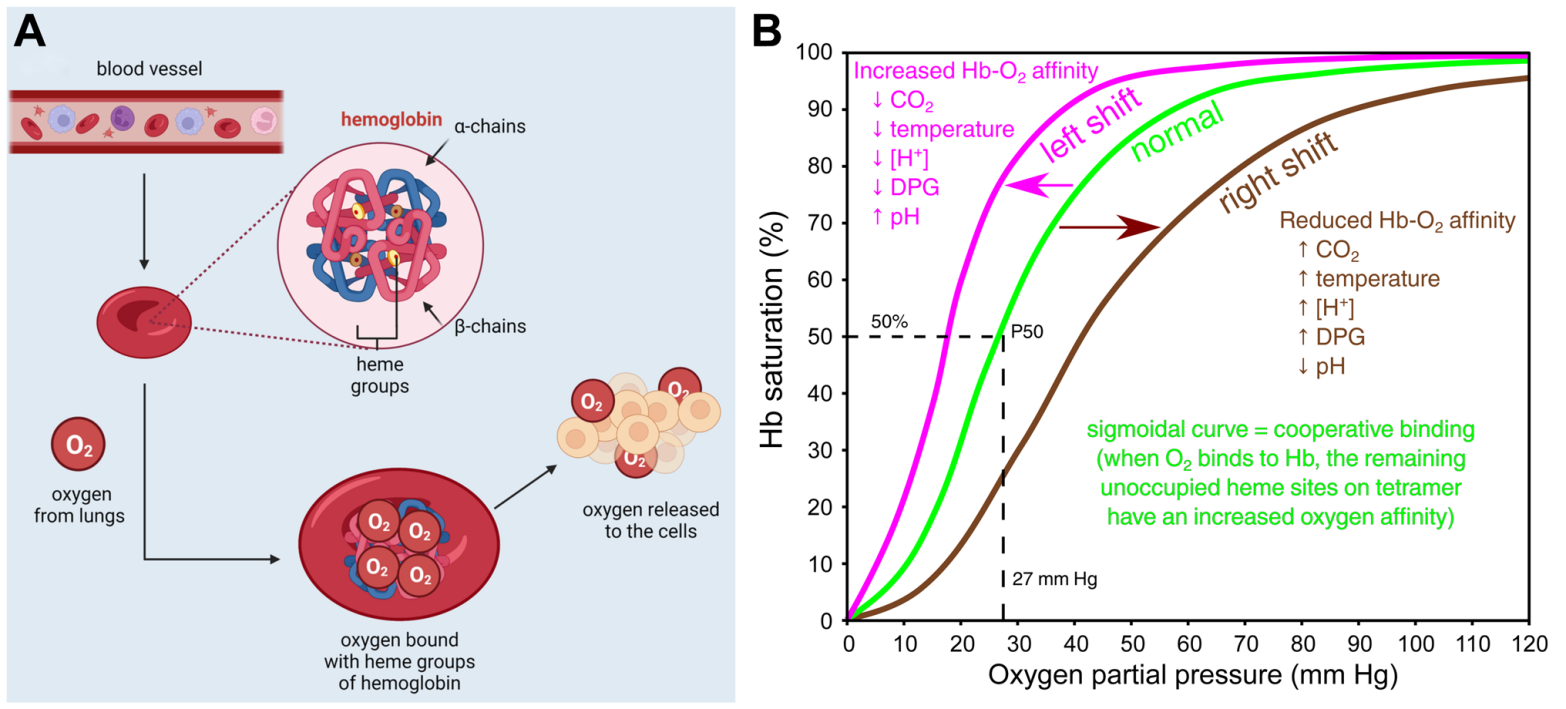
Oxygen transport by hemoglobin depends on many factors.** A** Simplified scheme of O_2_ transport by Hb. Emphasis is put on the quantity of heme groups in a single hemoglobin tetramer and thus a number of oxygen molecules bound and then released to cells of various tissues. **B** Oxygen–hemoglobin dissociation curve. Figure created using BioRender and Inkscape

Oxygen is an irreplaceable element that plays a crucial role in cellular energy [16]. O_2_ is obtained from the environment (atmospheric air) and transported to the lungs through the respiratory tract via the Hb within RBCs. A simplified graphical representation is depicted in Fig. 1A. Additionally to its role in oxygen and carbon dioxide transport, Hb enables the detoxification of reactive oxygen species (ROS) and some nitrogen compounds [17]. Oxygen saturation depends on the balance between the binding of O_2_ to Hb and the release of this chemical element from the tetramer. It also depends on many factors such as temperature, pH, pCO_2_ (partial pressure of carbon dioxide), oxygen tension, and concentration of 2,3-diphosphoglycerate (DPG) that is an allosteric effector stabilizing the low oxygen affinity form of hemoglobin [18]. The multi-subunit quaternary structure of hemoglobin influences oxygen affinity [19]. Even a small change in oxygen partial pressure (pO_2_) during blood flow from the lungs to the tissues can greatly affect the oxygen-binding or releasing by hemoglobin. This is confirmed by the classical sigmoidal shape of the oxygen equilibrium curve, which correlates with the oxygen saturation of hemoglobin across a range of oxygen pressures. In healthy adults, an oxygen partial pressure of ~27 mmHg corresponds to 50% hemoglobin saturation (P50). A right shift of the curve indicates that hemoglobin has a decreased affinity for oxygen. A shift to the left indicates increased hemoglobin affinity for oxygen and an increased reluctance to release oxygen. (Fig. 1B) [20]. The binding of an oxygen molecule to HbA increases the affinity of the remaining binding sites, but as soon as the first O_2_ is released, the oxygen affinity of the remaining globins drops significantly, which leads to a rapid release of the remaining oxygen, but only after the hemoglobin has reached tissues with a relatively low partial pressure of oxygen [14, 19]. In each of the heme groups, the binding of an oxygen molecule to a reduced iron atom (Fe^2+^) produces oxygenated hemoglobin called oxyhemoglobin that transports O_2_ from the lungs to the tissues. It is also worth mentioning that hemoglobin has a high tendency to undergo conformational changes, which contributes to the saturation of oxygen in the lungs and its release in the target tissues (conversion of oxyhemoglobin to deoxyhemoglobin). These changes are reversible and are aided, i.e., by 2,3-diphosphoglycerate and other allosteric effector molecules such as inositol hexaphosphate and bezafibrate [14]. However, outside the protective intact RBC environment (i.e., in a cell-free milieu), the hemoglobin tetramer is prone to rapidly disintegrate into protein subunits and is devoid of regulatory molecules like 2,3-diphosphoglycerate, methemoglobin reductase, superoxide dismutase, and catalase. This leads to decreased tissue oxygenation and the probability of conversion of Hb to methemoglobin (metHb) [10, 14].

### Mammalian hemoglobin-based oxygen carriers

Previous RBC substitutes have been divided into three categories: perfluorocarbon (PFC) emulsions, lipid or polymer-encapsulated mammalian hemoglobins, and polymerized or cross-linked mammalian hemoglobins [14]. PFC emulsions are linear, cyclic, or polycyclic hydrocarbons in which fluorine atoms replace hydrogen; these compounds are highly water-immiscible [1, 21] but are excessively toxic and do not provide oxygen in a controlled fashion [22]. Over the years, various generations of HBOCs have been developed. The initial generation of HBOCs consisted of Hb without red blood cell membranes and have demonstrated side effects such as fever, renal toxicity, decreased tissue perfusion, and even renal failure [1, 22]. This prompted the creation of the second generation of HBOCs by polymerizing Hb with aldehydes to reduce the risk of kidney toxicity. Unfortunately, its clinical trials revealed two other significant side effects, i.e., oxidative stress and nitric oxide scavenging.

As shown in Eq. 1, bound oxygen can contribute to the oxidation of the heme iron to Fe^3+^ and the generation of superoxide (O_2_^−^), which can cause oxidative damage to tissues [23]. Furthermore, the oxidized hemoglobin is devoid of the O_2_ transportability feature since it unable to bind O_2_ [24–27].

1 $$\mathrm{Hb}\left[{\mathrm{Fe}}^{2+}\right]{\mathrm{O}}_{2}\to \mathrm{Hb}\left[{\mathrm{Fe}}^{3+}\right]+{\mathrm{O}}_{2-}$$

2 $$\mathrm{Hb}\left[{\mathrm{Fe}}^{2+}\right]{\mathrm{O}}_{2}+\mathrm{NO}\to \mathrm{Hb}\left[{\mathrm{Fe}}^{3+}\right]+{\mathrm{NO}}_{3-}$$

Hemoglobin can also be oxidized by the gaseous hormone nitric oxide (NO), that is released into the bloodstream by cells to control vasal tension. As shown in Eq. 2, NO can also react with the oxygen bound to hemoglobin to form nitrate (NO_3_−) while also oxidizing the heme iron. Furthermore, the elimination of NO by this process induces vasoconstriction, which leads to dangerous side effects like hypertension [28, 29]. As a consequence, clinical trials have been halted in 2011 [30] and none of the listed polyHbs have been approved for general use by the U.S. Food and Drug Administration (FDA) or European Medicines Agency [15, 31–36].

The third generation of hemoglobin-based oxygen carriers can be categorized into chemically modified and encapsulated HBOC systems [14]. However, liposome-encapsulated hemoglobins showed a tendency to aggregate after several days in storage [37]. In contrast, the KaloCyte company constructed ErythroMer, a nanoencapsulated human hemoglobin that presented interactions with O_2_ and nitric oxide similar to the one in RBC [38]. The remarkable benefit of this carrier is able to bind and release oxygen molecules without constricting the blood vessels, but it is only stable after dissolving for a short period of time. However, the in vivo studies on ErythroMer are scarce, and this carrier has not yet been evaluated in human clinical trials [14, 39]. Currently, the greatest progress in research has been made in the case of modified hemoglobins, e.g., polyhemoglobins (polyHb) or combinations of Hb and fibrinogen in the form of polyHb-fibrinogen complexes [1].

Chemical modification of HBOCs involves hemoglobin cross-linking (intra- or intermolecular), surface modification (e.g., polyethylene glycol conjugation), or polymerization (e.g., with relevant redox enzymes). These types of RBC substitutes include, e.g., polyhemoglobin, polyHb-fibrinogen, Sangart’s Hemospan products, and Baxter’s HemAssist [14]. Polyhemoglobin was one of the first HBOCs to show therapeutic efficacy in patients with substantial trauma-related blood loss and who had undergone various types of surgery [18]. Another example is polyHb-fibrinogen, which plays an important role in replenishing not only erythrocytes but also platelets and clotting factors, allowing it to function as an oxygen carrier with thrombocyte-like activity [18, 40]. On the other hand, Sangart’s Hemospan products are examples of red blood cell substitutes created by macromeric bioconjugation with the use of polyethylene glycol (PEG), which allows for an increase in its stability and residence time in the vessels. The results of clinical trials revealed an increased risk of life-threatening bradycardia and also its administration in low doses has been associated with elevated levels of hepatic and pancreatic enzymes [41–43]. Another RBC substitute, i.e., HemAssist was produced by acylating hemoglobin with bis-(3,5-dibromosalicyl) fumarate but it failed clinical testing due to the increased patients’ mortality [44]. When it comes to encapsulated HBOC systems, these are substitutes containing hemoglobin enclosed in suitable particulate vehicles such as collodion (cellulose nitrate) or biodegradable polyethylene glycol polylactide (PEG-PLA) [14]. This type of HBOC utilizes bovine and human hemoglobin and includes the creation of polymersome-encapsulated Hb (PEH), although related in vivo studies are limited so far [39]. It is worth noting that in vivo and in vitro studies are ongoing for all encapsulated HBOC systems [14].

One of the attractive alternatives to previous HBOCs is a metalloprotein called erythrocruorin (Ec)—the red respiratory pigment found in annelids and crustaceans [45]. Ec possesses several advantages favoring its potential use as a red blood cell substitute. For instance, it has been demonstrated that the Ec of *Lumbricus terrestris* is highly stable, refractory to oxidation compared to adult hemoglobin (HbA), and may interact with NO in a manner that is distinct from that of mammalian Hbs [46]. The subsequent sections provide a more detailed description of erythrocruorins.

### Characteristics of erythrocruorin

Erythrocruorins are found in most annelids, some mollusks, and insects that live in a wide variety of environments. Even though the majority of erythrocruorins occur in marine annelids that live at relatively mild or cold temperatures, Ecs have also been identified in extremophiles like the polychaete *Alvinella pompejana* worms that occupy high-temperature thermal vents because they can survive under extreme conditions with very little oxygen [25]. Unfortunately, the difficulty of obtaining some Ec-containing extremophiles has limited the study of these atypical oxygen carriers [25].

Various types of Ecs have different shapes and sizes but their architecture is similar, based on electron microscopy data, these large extracellular multimeric proteins composed of 180–198 subunits are visible as hexagonal bilayers (HBLs) that form a double-layer structure with D6 symmetry [36, 47–50]. Each HBL contains multiple copies of five different Hb chains (A, B, C, D1, and/or D2) that assemble into dodecamers (e.g., A_3_B_3_C_3_D_3_) which are bound by trimers of four types of non-hemoglobin linkers (L1, L2, L3, and L4) that assemble the globins into the HBL. Moreover, the subunits are all stabilized by intramolecular and intermolecular covalent disulfide bonds and strong electrostatic or hydrophobic forces, which prevents the dissociation observed with mammalian hemoglobins [48, 51]. The molecular mass of Ecs is approximately 3.6×10^6^ Da and the sedimentation constant is approximately 60S. Instead of packaged in cells, the Ecs of many annelids and arthropods are freely dissolved in the blood. Despite the significant differences in structure between Ecs and mammalian Hb, Ecs bind and release oxygen in an allosteric fashion that is highly similar to the oxygen equilibrium curve shown in Fig. 1. However, some Ecs from marine worms tend to have similar high affinity to O_2_, e.g., ~28 mmHg for Ec from *Lumbricus terrestris* [47, 51].

The characteristics of Ecs vary depending on the organism from which they are derived. Some of these Ecs exhibit high oxygen affinity (e.g., Ecs of *Branchipolynoe symmytilida,* P50 = 0.9–1.4 mmHg or *Megascolides australis,* P50 = 2 mmHg), while others that are referred to as chlorocruorins (e.g., in *Eudistylia vancouverii,* P50 = 145mmHg and *Potamilla leptochaeta*, P50=155mmHg) are characterized by their low oxygen affinity due to the modified heme group (vinyl group at the second position of the porphyrin ring is replaced by a formyl group) [14, 52, 53]. In contrast, Ecs from terrestrial worms like *Glossocolex paulistus* Ec (GpEc) and *Lumbricus terrestris* (LtEc, P50=28 mmHg) have a more moderate oxygen affinity that is similar to human whole blood [46, 54, 55]. The subsequent paragraphs describe the structure and biophysical properties of the most thoroughly studied erythrocruorins that originate from two organisms (*Lumbricus terrestris* and *Arenicola marina*) and represent the first and second types of erythrocruorins, which can differ in some physicochemical properties (Table 1).

**Table 1:** Synopsis of properties of various erythrocruorins

**Erythrocruorin**	**Properties**
**LtEc**	Oxygen affinity similar to human blood [46, 54, 55] but 144 oxygen-binding hemes [56]; several Ca^2+^-binding sites; More compact structure to that of AmEc [47]
**AmEc**	156 oxygen-binding hemes [36]; potential anti-inflammatory, anti-bacterial, and anti-oxidant properties [36, 57–59]; effective as a preservation solution that is an additive to graft organ, Hemarina-M101 [60]; wide range of operating temperatures (4–37 °C) [61]
**Others**	Various oxygen affinity: either high (Ecs of *Branchipolynoe symmytilida* or *Megascolides australis* [25, 52, 53]), moderate (similar to human blood; Ecs from terrestrial worms like *Glossocolex paulistus* [46, 54, 55]), or low oxygen affinity (chlorocruorins with modified heme group [25, 52, 53])

**Fig. 2:**
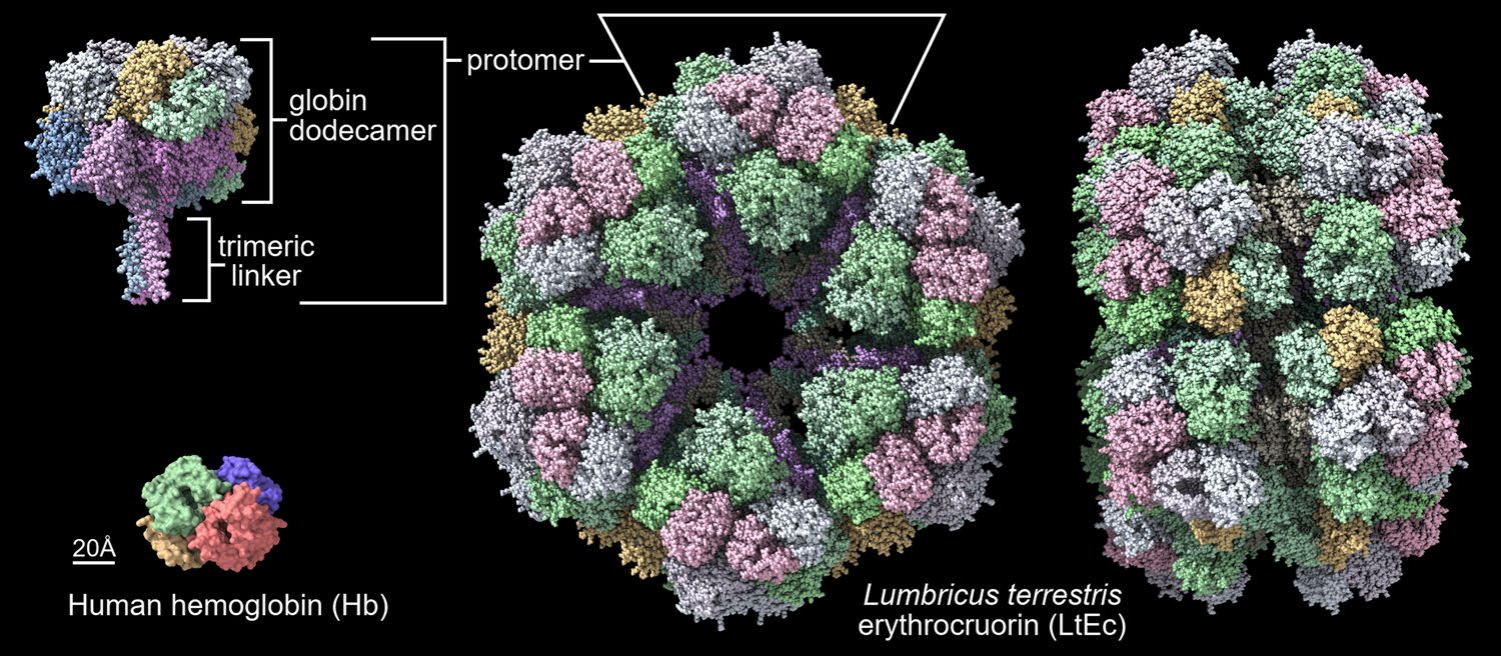
*Lumbricus terrestris* erythrocruorin. Two different orientations of LtEc are shown: along the molecular six-fold axis (center) and rotated 90° horizontally to emphasize the hexagonal bilayer (right). Dodecamer, linker, and protomer are highlighted. Human Hb is shown as a reference for size. Structures were acquired from Protein Data Bank (PDB) and visualized using ChimeraX v1.3. The PDB IDs were 4V93, 5M3L, and 2HHB, respectively for the entire LtEc molecule, LtEc protomer, and human Hb

The earthworm *Lumbricus terrestris* is thought to be native to Western Europe but it is now globally distributed in temperate to mild boreal climates. LtEc consists of 180 polypeptide chains which include 144 globin subunits and 36 linker chains [62]. Each globin subunit contains a single intramolecular disulfide bond and an oxygen-binding heme group. The amount of heme-based oxygen-binding sites in erythrocruorins is listed in Table 1. Interestingly, the structure of this globin resembles myoglobin more closely than mammalian Hb subunits [46]. Assembly of LtEc begins with the A, B, and C globins, which form intermolecular disulfide bonds to create a covalently linked ABC trimer (Fig. 2). In turn, the ABC trimer and D monomer (either D1 or D2) self-associate through electrostatic and hydrophobic interactions to form the ABCD tetramer [54]. The hexagonal bilayer is formed by trimers linked by disulfide bonds, which are formed by monomeric chains of globin D and globin A, B, and C chains, which are connected to heme-deficient linker chains. The formation of a full hexagonal LtEc structure is possible due to the presence of linker chains, which are also stabilized by intramolecular disulfide bridges. The three linker chains self-assemble to form the linker trimer (a triple-strand coiled coil) with several combinations of L1–L4 linker chains being possible during trimer formation. Three linker chains are present in 1/12 of a unit, each contributing one long amino-terminal helix to the observed helical coil. Twelve of these linker trimeric complexes give rise to a “spoke” of a triple-stranded coiled helical coil directed to the center of the complex. The resulting complex is a key stabilizing element as well as a scaffold for the 12 hemoglobin dodecamers [46, 51, 63]. Additionally, LtEc is also stabilized by several Ca^2+^-binding sites.

*Arenicola marina* is an organism found in tidal areas in a sulfide-rich environment with frequent fluctuations in environmental conditions [64]. AmEc exhibits general D6 symmetry and consists of 198 polypeptide chains that include 156 globin chains and 42 linker chains [36]. The hemoglobin subunits form the surface of the molecule, while the linker subunits are responsible for the core formation. The arrangement of AmEc subunits is very similar to that of LtEc [47]; however, there are also key differences. While the structure of the hemoglobin dodecamers is congruous in LtEc and AmEc, the arrangement of these dodecamers in two complexes is different due to the distribution of 36 linker subunits in the core of these Ecs. In type I erythrocruorins (e.g., LtEc), the vertices of the two hexagonal layers are partially shifted, with one layer being approximately 16° shifted from the other. With type II (e.g., AmEc), the vertices are essentially eclipsed [65]. Moreover, the architecture of type I erythrocruorin allows a more compact structure than that of type II and it has been included in Table 1. This is due to the fact that in type I the triple-stranded coiled coils (of protomer) are broken into longer (approx. 45Å) and shorter (approx. 20Å) coiled coil with a disjointed connection, while type II is characterized by a continuous long ~70Å coiled coil [47]. Comparison of Ecs with traditional HBOCs is summarized in Table 2 and will be invoked multiple times in subsequent sections.

**Table 2:** Comparison of erythrocruorins with traditional HBOCs

	**Erythrocruorins**	**Traditional HBOCs**
**Heme-based oxygen-binding sites**	144 (LtEc) [63]; 156 (AmEc) [36]	4 [56]
**Molecular weight (kDa)**	~3600 (LtEc and AmEc) [65, 66]	64 (HbA) [46]
**Disulfide bonds occurrence**	Present, reducing the Ecs susceptibility to dissociation [67]	Lacking in mammalian Hb [46]
**Protein stability after in vivo transfusion**	Decent stability [68]	Instability caused by high osmotic stress [34, 69]
**Toxicity after in vivo transfusion**	Lower rate of nitric oxide dioxygenation reaction; less amount of removed NO [70]	Higher rate of nitric oxide dioxygenation reaction; abundant removal of NO [70]
	Greater oxidation resistance due to high redox potential [46]	Risk of metHb formation due to the high heme oxidation rate [46]
**Redox potential (mV)**	+63 (LtEc) [46]; +112 (AmEc) [46]	−50 (HbA) [46]
**Oxidation rate (h**^**−1**^**)**	2.0×10^−4^ (LtEc) [71]; 4×10^−3^ (Ecs on average) [72]	4.3×10^−2^ (HbA) [72]
**Cooperativity** **(Hill coefficient)**	3.74 (LtEc) [70]; 2.4 ± 0.01 (LtEc) [71]; 2.2 ± 0.2 (LtEc-PAA) [71]; 2.4 ± 0.1 (LtEc-PAA-EDA) [71]	2.43 (HbA) [70]; 2.45 (bovine [b] Hb) [70]; 0.84 (PolybHb 40:1*) [70]; 0.87 (PolybHb 50:1*) [70]
**O**_**2**_** affinity (P50) (mmHg)**	~28 (LtEc) [73]; ~2.6 (AmEc, physiological conditions of *Arenicola marina*) [65]; ~7 (AmEc, under human physiological conditions) [65]	~11 (HbA) [65]; ~26 (RBC) [74]
**Circulation half-life (h)**	14 (LtEc) [71]; 12 (AmEc) [25]	45 (HbA) [62]
**Alkaline dissociation**	Native LtEc dissociates at pH 9 while subunits remain resistant up to pH 10 [71]; AmEc dissociates at a slightly alkaline pH (between 7 and 8) [65]	Most of the Hb molecules are dimers at pH 8, with some coexisting tetramers (but no monomers). When pH 9 is reached, most of the Hb molecules are monomers while only a few are tetramers [75]
**Oncotic pressure** **(mmHg)**	14 (LtEc) [70]	1-5 (polyHb) [70]
**Vasal tension**	Presumable slight vasodilation (LtEc) [46]	Vasoconstriction (HbA) [76]
**Modification aims**	Purity and stability improvements [55, 77]	Cross-linking, surface modification, and polymerization [14]
**Modification outcomes**	Unaffected O_2_ transportability, no severe side effects after transfusion of modified Ecs [71]	Promising study results regarding polyHb and polyHb-fibrinogen complex; existing side effects in others [18, 34]
**Synopsis of oxygen carriers’ advantages**	Freely dissolved in the annelids’ blood (Ecs are acellular) [46, 48]; More oxygen-binding hemes compared to HbA [56]; AmEc is not scavenged by haptoglobin [46]; Superior O_2_ delivery due to the large molecule size [77]; Reduced susceptibility to dissociation at low concentrations compared to mammalian Hb [46]; Longer storage time compared to RBC (at least 7 days at 37 °C) [78]; Significant reduction of nitric oxide dioxygenation [46]; High redox potential, high stability, and lower toxicity [77];	RBC can provide more same sustained O_2_ transport than Ecs [63, 64, 79]; New generation of HBOCs has a longer half-life than Ecs [46, 80]

^*^Bovine Hb polymerized at two different molar ratios of glutaraldehyde:Hb

### The potential use of erythrocruorin in medicine

Due to its structural and functional properties, erythrocruorin is a promising red blood cell substitute and has been undergoing preclinical transfusion studies in rodents for many years [78, 81, 82]. Deoxygenated LtEc can be stored at 37 °C for at least 7 days without losing its function, which is a significant improvement over donated RBCs that must be continuously refrigerated (Table 1) [78]. This is extremely beneficial for military operations or in third world countries. Various experiments involving the injection of small amounts of LtEc and AmEc into laboratory animals (mainly mice and rats) have shown that these compounds do not seem to cause side effects in vivo in rodents; however, only a few parameters have been investigated [65, 81]. The half-life of LtEc is approximately 14 hours (similar to HBOCs with half-lives of approximately 19 h), which is shorter compared to RBCs (months), so it is worth noting that it cannot provide the same sustained O_2_ transport (Table 1) [63, 64, 79]. However, the work of Teruyuki Komatsu’s group gave insight into Hemoglobin-Albumin clusters as RBC substitutes and shows the advantage of new generations of HBOC over Ecs. The research of this scientific group indicated that the HbBv-HSA_3_ cluster has sufficient preclinical safety as a potential alternative material used for RBC transfusions, and its circulation half-life was 18.5 hours [80]. In vitro experiments also indicated that AmEc is not scavenged by haptoglobin, a serum protein that strongly binds to free HbA and clears it from the bloodstream [46]. The circulation half-life of AmEc, LtEc, and HbA is mentioned in Table 2. Nevertheless, cell-free hemoglobin that is released from aged or damaged RBCs dissociates in dimers that have a half-life of only a few hours [13]. Since Ecs have much larger structures their rapid renal uptake might be prevented making their half-life longer, especially if they are cross-linked with glutaraldehyde [66] although the precise mechanism is yet unclear.

Regarding LtEc, it was shown that multiple injections of erythrocruorin into hamsters did not trigger an immune response, which was determined by checking IgE and IgG2 antibodies [65]. In addition, it was observed that LtEc caused slight vasodilation instead of the vasoconstriction observed with other HBOCs (Table 2) [46]. It is suggested that owing to the naturally occurring amino acids such as phenylalanine and tryptophan residues within the heme pockets, LtEc may have a significantly reduced nitric oxide dioxygenation. This is a very important advantage of Ecs over HbA because it means that LtEc can avoid the increase in blood pressure and systemic hypertension observed with previous blood substitutes [46]. Additional experiments in hamsters showed that LtEc is effective in maintaining systemic hemodynamics, preserving blood gas parameters, and delivering O_2_, which prevents tissue anoxia that results from extreme anemia (11% hematocrit). Compared to other plasma expanders, LtEc presented superior O_2_ delivery due to the large molecule size, O_2_ binding characteristics, high viscosity, stability, and long circulating half-life [77]. LtEc oncotic pressure is slightly reduced compared to the norm of human whole blood (19–24.5 mmHg). The oncotic pressure is important because it determines the direction of fluid movement in the body; hence, the relative concentration of ions and protein in the solvent [70]. Stability as well as toxicity after in vivo transfusion are described and compared to HBOCs in Table 2. A study by Hirsch et al. indicated that LtEc could be safely transfused into rats and mice, without any harmful interaction between LtEc and plasma components [81]. With regard to large projects on LtEc, at least one is ongoing (ID: 1R15HL133880-01A1) based on the National Institutes of Health (reporter.nih.gov).

Like LtEc, AmEc has been successfully transfused into rodents and it did not elicit an immune response or changes in blood pressure [25]. One study showed that transfusion into mice did not cause any changes in the general behavior of those animals or their growth [65]. In contrast to LtEc, AmEc dissociation from the hexagonal bilayer into dodecamers is quick when exposed to low ionic strength in human plasma. Nonetheless, transfusions of both LtEc and AmEc have been shown to be effective in maintaining oxygen delivery in rodents [25, 65, 77, 81]. Potential anti-inflammatory, anti-bacterial, and anti-oxidant properties of AmEc have also been demonstrated. All of these potential properties are listed in Table 1. This O_2_ carrier is also effective as a preservation solution that is an additive to graft or organ (e.g., heart, liver, lung, or kidney) under perfusion conditions [36, 57–59]. Additionally, studies indicate that AmEc may be a potential therapeutic agent for the healing and regeneration of periodontal wounds [83]. A recent in vitro study by Dare et al. proved the positive effect of AmEc in the reduction of amanitin-induced toxicity in a cell-based model of the liver. The erythrocruorin of *Arenicola marina* not only increased the viability of the human hepatocyte carcinoma cell line HepaRG but also restored the viability of these cells and decreased the production of mitochondrial ROS after exposure to α- and β-amanitin. The hepatoprotective effect of AmEc can be attributed to the ability of this compound to deliver oxygen [84]. In turn, the results from Batool et al. study showed the anti-inflammatory and anti-microbial potential of AmEc; no signs of cytotoxicity were observed in oral epithelial cells treated with AmEc. This hemoglobin may decrease the level of pro-inflammatory markers (e.g., tumor necrosis factor alpha [TNF-α], nuclear factor kappa B [NF-κΒ], and receptor activator of NF-κB ligand [RANKL] in *P. gingivalis*-lipopolysaccharide-stimulated and *P. gingivalis*-infected Ecs) or increase the level of healing mediators (e.g., platelet-derived growth factor isoform BB [PDGF-BB], transforming growth factor β1 [TGF-β1], interleukin-10 [IL-10]), and immune modulators. The treatment reduced inflammatory cell infiltration (ICI) and improved bone healing. Moreover, the anti-bacterial activity of this compound on *P. gingivalis* biofilm formation, as well as the reduction of bacterial growth, were demonstrated [83]. Clinical trial data from the National Institutes of Health (clinicaltrials.gov) indicate one currently recruiting phase 3 trial (identifier: NCT04181710) which evaluates the effectiveness of an AmEc-based medical solution called HEMO2life^®^. This device is an oxygenation solution used for organ preservation and has recently obtained the CE marking, which proves its quality in terms of safety and clinical benefits based on EU regulations [85]. Hemarina-M101 is a new non-toxic therapeutic oxygen carrier developed from *Arenicola marina* blood. Lower partial pressure is required to saturate some of the typical O_2_-binding sites with blood, allowing oxygen to be released solely to hypoxic cells. The advantages of this carrier are non-immunogenicity, a wide range of operating temperatures (4–37 °C), and anti-oxidant properties due to the activity of superoxide dismutase [65]. Recent in vivo studies also presented no effect on heart rate or mean arterial pressure, and lower nitric oxide binding properties [60]. The research results indicate that Hemarina-M101 may have a protective effect against ischemic reperfusion injury that is important after transplantation [60].

In addition to erythrocruorin, two major oxygen-binding proteins that are beneficial in medicine are worth mentioning: hemocyanins (Hcs) and hemerythrins (Hrs) [45, 86]. For the former, two main superfamilies can be distinguished, i.e., arthropod Hcs and mollusk Hcs, which vary in terms of their structural appearance. At present, due to their high therapeutic potential, Hcs are under research in various directions, including their use as bio-adjuvants, immune stimulants, and vaccines [45, 87, 88]. Hcs have also displayed positive outcomes in studies conducted in vivo. According to its biochemical and immunological properties in the rat *in vivo* models, Hrs were found to behave in a manner similar to hemoglobins, which makes them another promising research direction [89].

### Advantages of erythrocruorins over other HBOCs

Many hemoglobin-based oxygen carriers have critical biophysical limitations and induce severe side effects after in vivo transfusion. One of them is protein instability caused by the generation of high vascular osmotic stress. The second negative aspect is the toxicity of these compounds due to the high rate of heme oxidation [34, 56, 69] and the removal of nitric oxide [90]. The latter is caused by dioxygenation, which directly contributes to pro-inflammatory conditions and cardiovascular problems due to vasoconstriction [3, 91]. On the other hand, the auto-oxidation of the oxygen-bound heme threatens the formation of a metHb that is capable of generating ROS with pro-inflammatory potential. These disadvantages can be observed after removing RBCs from mammals and using them in the production of HBOCs. For this reason, extracellular hemoglobins (such as erythrocruorins) from organisms that lack red blood cells may constitute a better source of red blood cell substitution [46, 56].

The fact that erythrocruorins, unlike most hemoglobins, are freely dissolved in the annelids’ blood is a definite advantage. Furthermore, Ecs are resistant to harsh conditions owing to their structural and functional features [46, 48]. Another indispensable structural feature of erythrocruorins is the presence of covalent disulfide bonds and the electrostatic or hydrophobic forces that hold them together. In contrast to mammalian hemoglobins, the presence of intermolecular disulfide bonds reduces the susceptibility of Ecs to dissociation at low concentrations [46]. Furthermore, the structure of Ec allows superior binding ability compared to that of Hb; erythrocruorins possess many heme-based oxygen transport sites. For example, each LtEc molecule contains 144 oxygen-binding hemes, while HbA has only four [56]. Moreover, Ecs have a high redox potential, the value of which is presented in Table 2 [27]; this influences the heme’s tendency to reduce and form ferrous hemoglobin. It also contributes to their naturally occurring high stability and lower toxicity [77]. Reduced toxicity is due to the fact that these red blood cell substitutes are resistant to oxidation and do not remove NO to such a great extent. The latter feature stems from the fact that Ecs have smaller heme pockets that prevent the simultaneous binding of O_2_ and NO so that nitrate cannot be formed in comparison to other HBOCs [56, 70, 78]. Another aspect that is typically measured using the Hill coefficient (*n*) [92] is cooperativity, often described together with the oxygen affinity that is mentioned earlier in the Introduction. The binding of the first O_2_ leads to a gradual increase in oxygen affinity until all Hb binding sites are filled in a cooperative manner [93]. Hill coefficient *n*>1 represents positive cooperativity (allosteric interactions between subunits), while *n*=1 indicates a non-cooperative O_2_ binding [25]. Negative cooperativity (*n*<1) can be observed when the affinity of the enzyme for subsequent ligands decreases once the first ligand molecule is bound. Promising data are available in the literature, suggesting higher cooperativity of LtEc relative to HbA (*n*=3.74 vs. *n*=2.43) [70]. At the same time, LtEc has the highest Hill coefficient when compared to erythrocruorins from *Amynthas gracilis* (AgEc, n=2.16), *Eudrilus eugeniae* (EeEc, *n*=1.96), *Eisenia fetida* (EfEc, *n*=2.39), *Eisenia hortensis* (EhEc, *n*=1.97), and *Eisenia veneta* (EvEc, *n*=2.13) [25]. As for traditional HBOCs, some modifications of native Hb can drastically decrease cooperativity [94].

Another advantage of erythrocruorins is related to many modifications that have been implemented over the years, allowing the production of products with enhanced stability. So far, the main methods used to purify these extracellular hemoglobins are tangential flow filtration (TFF), anion-exchange purification (AEX), and immobilized metal affinity chromatography (IMAC) [55]. TFF involves using a filter to separate or isolate particles of specific sizes. The filtrate is discharged across, creating a differential of pressure, and the sample flows parallel to the filter and is recirculated multiple times. Using TFF, purified Ec was obtained with high yield and favorable biophysical properties, presenting no side effects when transfused to laboratory animals [55, 70]. The AEX approach that separates proteins based on a surface charge is more appropriate for the purification of Ecs than the IMAC method that is based on the interaction of amino acids with metals, since the latter method caused oxidation and dissociation of Ecs [73]. Even though the Ecs purified with TFF and other methods may not be 100% pure, they have been safely transfused into animals without side effects caused by impurities [70]. As mentioned above, erythrocruorins are relatively stable proteins; however, in order to increase their effectiveness as red blood cell substitutes, the resistance to high temperatures over a long period of storage should be refined. A study by Spivack et al. presented that the cross-linking of Ec with polyacrylic acid (PAA) and ethylenediamine (EDA) can be useful in enhancing the stability of this hemoglobin while maintaining its natural oxygen transport capacity, and even increasing its affinity to oxygen [95]. Conjugation with PEG allows for a prolongation of the LtEc half-life in the circulation (up to four times longer than that of non-PEGylated LtEc). However, this method resulted in faster oxidation of this compound in vivo, as can be seen from cooperativity values (Hill coefficient) in Table 2 [68]. On the other hand, covalent cross-linking of LtEc with glutaraldehyde (gLtEc) resulted in an increase of the melting point (from the initial *T*_*m*_=57 °C to *T*_*m*_=68 °C, where the glutaraldehyde-to-heme ratio was 128:1) and a reduction in the dissociation of subunits at alkaline pH. Moreover, the LtEc modified using this approach was still capable of transporting oxygen, with a higher affinity for this chemical element and only with a slight increase in the rate of oxidation [66]. It is also worth mentioning that both LtEc and AmEc have been successfully lyophilized and resuspended [23, 96]. A comparison of erythrocruorins with traditional HBOCs is summarized in Table 2, where there can be seen, among other things, the rate of oxidation of Ecs as well as, modification aims and outcomes for these compounds.

## Conclusions

As the availability of blood decreases and its price rises, the demand for red blood cell substitutes is unquestionable. Artificial oxygen carriers are versatile agents that can be used in patients with blood loss associated with trauma and transplantation. It is crucial to understand the action mechanisms of hemoglobin since HBOCs use this protein to carry oxygen. Many different red blood cell substitutes have been tested for effectiveness. However, following transfusion, a large proportion of them have presented key limitations such as protein instability and toxicity due to NO scavenging and oxidation to methemoglobin. The use of erythrocruorin might help to overcome these limitations. This metalloprotein could therefore be considered a promising RBC substitute. The major research focus was drawn on erythrocruorin derived from *Lumbricus terrestris* and *Arenicola marina*. These erythrocruorins exhibit different structural features that determine their properties; however, both can be used as oxygen carriers. The development of methods of purification (TFF, AEX, IMAC) and stabilization (PEGylation, PAA, and EDA cross-linking) could enable the use of these macromolecular structures in the clinic, which would increase the accessibility to artificial RBC resources.

## Supplementary Information

Below is the link to the electronic supplementary material.

Supplementary file1 (PDF 441 KB)

## Data Availability

The datasets generated during and/or analyzed during the current study are available in the PDB repository, https://www.rcsb.org/.
