# Supplementary material for: The artificial oxygen carrier erythrocruorin—characteristics and potential significance in medicine
Source: J Mol Med (Berl). 2023 Jul 18;101(8):961–72. doi: 10.1007/s00109-023-02350-3 (PMC10400677; doi:10.1007/s00109-023-02350-3)
Supplement: Supplementary file 1 — Supplementary file1 (PDF 441 KB) [file 109_2023_2350_MOESM1_ESM.pdf]

Adis Bohn Stafleu van Loghum palgrave macmillan Springer

# Change of authorship request form (pre-acceptance)

## Please read the important information on page 4 before you begin

This form should be used by authors to request any change in authorship including changes in corresponding authors. Please fully complete all sections. Use black ink and block capitals and provide each author's full name with the given name first followed by the family name.

Please note: In author collaborations where there is formal agreement for representing the collaboration, it is sufficient for the representative or legal guarantor (usually the corresponding author) to complete and sign the Authorship Change Form on behalf of all authors.

| |
|---|
| **Manuscript ID no. in case of unpublished manuscript: JMME-D-22-00490R2** |
| **DOI in case of published manuscript:** ***not applicable*** |
| **ISBN (for books):** ***not applicable*** |

**Section 1: Please provide the current title of manuscript**
(For journals: Please provide the manuscript ID, title and/or DOI if available.)
(For books: Please provide the title, ISBN and/or DOI if available.)

Title: Erythrocruorin as a pioneering artificial oxygen carrier with potential significance in medicine

**Section 2: Please provide the previous authorship, in the order shown on the manuscript before the changes were introduced. Please indicate the corresponding author by adding (CA) behind the name.**

| | First name(s) | Family name | ORCID or SCOPUS id, if available |
|---|---|---|---|
| 1st author | Weronika | Kruczkowska | 0000-0003-1699-8912 |
| 2nd author | Mateusz | Kciuk | 0000-0002-8616-3825 |
| 3rd author | Zbigniew | Pasieka | 0000-0001-8931-0213 |
| 4th author | Karol | Kłosiński | 0000-0003-0962-228X |
| 5th author | Elżbieta | Płuciennik | 0000-0002-6682-0402 |
| 6th author | Klaudia | Waszczykowska | 0000-0003-3102-3419 |
| 7th author | Damian | Kołat | 0000-0002-1086-3796 |

Please use an additional sheet if there are more than 7 authors.

---
SPRINGER NATURE Springer Nature is one of the world's leading global research, educational and professional publishers, created in May 2015 through the combination of Nature Publishing Group, Palgrave Macmillan, Macmillan Education and Springer Science+Business Media.
---

# Change of authorship request form (pre-acceptance)

| | First name(s) | Family name | ORCID or SCOPUS id, if available |
|---|---|---|---|
| 8th author | Żaneta | Kałuzińska-Kołat (CA) | 0000-0002-2335-3293 |

Adis Bohn Stafleu van Loghum palgrave macmillan Springer

# Change of authorship request form (pre-acceptance)

**Section 3: Please provide a justification for change. Please use this section to explain your reasons for changing the authorship of your manuscript, e.g. what necessitated the change in authorship? Please refer to the (journal) policy pages for more information about authorship. Please explain why omitted authors were not originally included and/or why authors were removed on the submitted manuscript.**

In order to accomplish the second round of revisions (required by Reviewer 2, since Reviewer 1 accepted the paper after the first round), and due to the vague changes suggested by Reviewer 2, we decided that the best solution would be to invite one new co-author, who is an expert in the field.

Jacob Elmer is a well-known scientist in the field of erythrocruorins, and were kindly asked for critical review of the paper's content. The improvements over the last manuscript version are implemented thanks to a close cooperation of all authors.

The addition of new co-author to the previous authorship list is in line with conversation between corresponding author Żaneta Kałuzińska-Kołat and Managing Editor Friederike Hezel-Frommer (on behalf of Editor-In-Chief Ari Waisman).

All required details of Jacob Elmer (affiliation, ORCID, contribution) are included in this manuscript.

Institutional e-mail of Jacob Elmer is: jacob.elmer@villanova.edu.

**Section 4: Proposed new authorship. Please provide your new authorship list in the order you would like it to appear on the manuscript. Please indicate the corresponding author by adding (CA) behind the name. <u>If the corresponding author has changed, please indicate the reason under section 3.</u>**

| | First name(s) | Family name (this name will appear in full on the final publication and will be searchable in various abstract and indexing databases) |
|---|---|---|
| 1st author | Weronika | Kruczkowska |
| 2nd author | Mateusz | Kciuk |
| 3rd author | Zbigniew | Pasieka |
| 4th author | Karol | Kłosiński |
| 5th author | Elżbieta | Płuciennik |
| 6th author | Jacob | Elmer |
| 7th author | Klaudia | Waszczykowska |

Please use an additional sheet if there are more than 7 authors.

---
SPRINGER NATURE Springer Nature is one of the world's leading global research, educational and professional publishers, created in May 2015 through the combination of Nature Publishing Group, Palgrave Macmillan, Macmillan Education and Springer Science+Business Media.
---

# Change of authorship request form (pre-acceptance)

| | First name(s) | Family name (this name will appear in full on the final publication and will be searchable in various abstract and indexing databases) |
|---|---|---|
| 8th author | Damian | Kołat |
| 9th author | Żaneta | Kałuzińska-Kołat (CA) |

---
SPRINGER NATURE Springer Nature is one of the world's leading global research, educational and professional publishers, created in May 2015 through the combination of Nature Publishing Group, Palgrave Macmillan, Macmillan Education and Springer Science+Business Media.
---

Adis Bohn Stafleu van Loghum palgrave macmillan Springer

# Change of authorship request form (pre-acceptance)

**Section 5: Author contribution, Acknowledgement and Disclosures. Please use this section to provide a new disclosure statement and, if appropriate, acknowledge any contributors who have been removed as authors and ensure you state what contribution any new authors made (if applicable per the journal or book (series) policy). Please ensure these are updated in your manuscript - after approval of the change(s) - as our production department will not transfer the information in this form to your manuscript.**

**New acknowledgements:**

*Not applicable*

**New Disclosures (financial and non-financial interests, funding):**

*Not applicable*

**New Author Contributions statement (if applicable per the journal policy):**

WK, DK, and ŻKK conceptualized the article. ŻKK and DK supervised the article. MK, ŻKK, DK, and KW visualized the figures and prepared a table. WK wrote the original draft based on a literature search. WK, MK, ZP, KK, EP, JE, KW, DK, and ŻKK reviewed and edited the article. All authors have read and agreed to the published version of the manuscript.

State 'Not applicable' if there are no new authors.

---
SPRINGER NATURE Springer Nature is one of the world's leading global research, educational and professional publishers, created in May 2015 through the combination of Nature Publishing Group, Palgrave Macmillan, Macmillan Education and Springer Science+Business Media.
---

Adis Bohn Stafleu van Loghum palgrave macmillan Springer

# Change of authorship request form (pre-acceptance)

**Section 6: Declaration of agreement. *All* authors, unchanged, new and removed *must* sign this declaration.**
(NB: Please print the form, sign and return a scanned copy. Please note that signatures that have been inserted as an image file are acceptable as long as it is handwritten. Typed names in the signature box are unacceptable.) *** Please delete as appropriate. Delete all of the bold if you were on the original authorship list and are remaining as an author.**

| | First name | Family name | | Signature | Affiliated institute | Date |
|---|---|---|---|---|---|---|
| 1st author | Weronika | Kruczkowska | I agree to the proposed new authorship shown in section 4 | Weronika Kruczkowska | Faculty of Biomedical Sciences, Medical University of Lodz, Zeligowskiego 7/9, 90-752, Lodz, Poland | 03/17/2023 |
| 2nd author | Mateusz | Kciuk | I agree to the proposed new authorship shown in section 4 | Mateusz Kciuk | Department of Molecular Biotechnology and Genetics, University of Lodz, Banacha 12/16, 90-237, Lodz, Poland<br>Doctoral School of Exact and Natural Sciences, University of Lodz, Banacha 12/16, 90-237, Lodz, Poland | 03/22/2023 |
| 3rd author | Zbigniew | Pasieka | I agree to the proposed new authorship shown in section 4 | [illegible] | Department of Experimental Surgery, Medical University of Lodz, Narutowicza 60, 90-136, Lodz, Poland | 03/21/2023 |
| 4th author | Karol | Kłosiński | I agree to the proposed new authorship shown in section 4 | Karol Kłosiński | Department of Experimental Surgery, Medical University of Lodz, Narutowicza 60, 90-136, Lodz, Poland | 03/18/2023 |
| 5th author | Elżbieta | Płuciennik | I agree to the proposed new authorship shown in section 4 | Elżbieta Płuciennik | Department of Functional Genomics, Medical University of Lodz, Zeligowskiego 7/9, 90-752, Lodz, Poland | 03/17/2023 |
| 6th author | Jacob | Elmer | I agree to the proposed new authorship shown in section 4 **and the addition of my name to the authorship list.** | JE | Department of Chemical and Biological Engineering, Villanova University, Villanova, Pennsylvania, USA | 03/20/2023 |
| 7th author | Klaudia | Waszczykowska | I agree to the proposed new authorship shown in section 4 | Klaudia Waszczykowska | Department of Functional Genomics, Medical University of Lodz, Zeligowskiego 7/9, 90-752, Lodz, Poland | 03/17/2023 |

Please use an additional sheet if there are more than 7 authors.

---
SPRINGER NATURE Springer Nature is one of the world's leading global research, educational and professional publishers, created in May 2015 through the combination of Nature Publishing Group, Palgrave Macmillan, Macmillan Education and Springer Science+Business Media.
---

# Change of authorship request form (pre-acceptance)

| | First name | Family name | | Signature | Affiliated institute | Date |
|---|---|---|---|---|---|---|
| 8th author | Damian | Kołat | I agree to the proposed new authorship shown in section 4 | Damian Kołat | Department of Experimental Surgery, Medical University of Lodz, Narutowicza 60, 90-136, Lodz, Poland | 03/17/2023 |
| 9th author | Żaneta | Kałuzińska-Kołat | I agree to the proposed new authorship shown in section 4 | Żaneta Kałuzińska-Kołat | Department of Experimental Surgery, Medical University of Lodz, Narutowicza 60, 90-136, Lodz, Poland | 03/17/2023 |

# Change of authorship request form (pre-acceptance)

## Important information. Please read.

- Please return this form, fully completed, to Springer Nature. We will consider the information you have provided to decide whether to approve the proposed change in authorship. We may choose to contact your institution for more information or undertake a further investigation, if appropriate, before making a final decision.

- By signing this declaration, all authors guarantee that the order of the authors are in accordance with their scientific contribution, if applicable as different conventions apply per discipline, and that only authors have been added who made a meaningful contribution to the work.

- Please note, we cannot investigate or mediate any authorship disputes. If you are unable to obtained agreement from all authors (including those who you wish to be removed) you must refer the matter to your institution(s) for investigation. Please inform us if you need to do this.

- If you are not able to return a fully completed form within **30 days** of the date that it was sent to the author requesting the change, we may have to withdraw your manuscript. We cannot publish manuscripts where authorship has not been agreed by all authors (including those who have been removed).

- Incomplete forms will be rejected.
